# Low tristetraprolin expression promotes cell proliferation and predicts poor patients outcome in pancreatic cancer

**DOI:** 10.18632/oncotarget.7397

**Published:** 2016-02-15

**Authors:** Zi-Ran Wei, Chao Liang, Dan Feng, Ya-Jun Cheng, Wei-Min Wang, De-Jun Yang, Yue-Xiang Wang, Qing-Ping Cai

**Affiliations:** ^1^ Department of Gastro-Intestine Surgery, Changzheng Hospital, Second Military Medical University, Shanghai, China; ^2^ Department of Oncology, Changhai Hospital, Second Military Medical University, Shanghai, China; ^3^ SIBS (Institute of Health Sciences)-Changzheng Hospital Joint Center for Translational Medicine, Institute of Health Sciences, Shanghai Changzheng Hospital, Institutes for Translational Medicine (CAS-SMMU), Key Laboratory of Stem Cell Biology, Institute of Health Sciences, SIBS, Chinese Academy of Sciences/Shanghai JiaoTong University School of Medicine, Collaborative Innovation Center of Systems Biomedicine, Shanghai, China

**Keywords:** tristetraprolin, pancreatic cancer, IL-6, Pim-1, prognosis

## Abstract

Tristetraprolin (also known as TTP, TIS11, ZFP36, and Nup475) is a well-characterized tumor suppressor that is down-regulated in several tumor types. In the current study, we found that TTP expression was markedly reduced in pancreatic cancer samples as compared to matched normal tissues. Low TTP level was associated with age (P=0.037), tumor size (P=0.008), tumor differentiation (P=0.004), postoperative T stage (pT stage, P<0.001), postoperative N stage (pN stage, P=0.008) and TNM stage (P<0.001). Moreover, low TTP expression predicted reduced survival rates and poor patient outcome. We also found that TTP impairs pancreatic cancer cell proliferation both *in vivo* and *in vitro*. Fluorescence Activated Cell Sorting (FACS) assay showed that TTP over-expression both increases apoptosis and decreases proliferation in pancreatic cancer cells. RNA-sequencing analysis showed that TTP over-expression downregulates several tumor-related factors, including Pim-1 and IL-6. Our findings indicate that TTP could serve as a potential prognostic indicator in pancreatic cancer.

## INTRODUCTION

Dysregulation of oncoproteins, cytokines, and inflammatory mediators can influence the initiation and progression of cancer [1]. Cytokines play an important role in the development and tumorigenesis of pancreatic cancer, which is the fourth leading cause of cancer-related death globally [2]. Interleukin-6 (IL-6), a vital inflammation-related cytokine that is enhanced by hypoxia and K-ras, and in turn contributes to the formation of the tumor microenvironment, has been implicated in pancreatic cancer angiogenesis and metastasis [3]. Ebrahimi, *et al*. reported that IL-6 level is an independent prognostic factor in pancreatic cancer [4]. Downregulation of Pim-1, a member of the serine/threonine protein kinase family and an oncogene involved in cell survival, proliferation, differentiation and tumorigenesis [5], reduces tumorigenicity and increases apoptosis in pancreatic cancer cells [6]. Pim-1 also enhances NFATc1 activity [7], which increases proliferation and anchorage-independent growth in pancreatic cancer [8].

Expression of these inflammation- and tumor-related proteinsmay be influenced by RNA-binding proteins (RNA-BPs) that promote mRNA degradation. RNA-BPs regulate mRNA stability by binding AU-rich elements (AREs), sequences located within the 3′-untranslated region (3′-UTR) of many transcripts [9,10]. Therefore, RNA-BPs may represent prognostic indicators and/or potential therapeutic targets for cancer treatment.

Tristetraprolin (TTP) is a well-characterized RNA-BP containing two zinc fingers necessary for TTP recognizes ARE sequences through adjacent AUUUA-binding site [11, 12]. TTP is frequently suppressed in human cancers, likely influencing patient outcomes [13]. The mRNA transcripts of IL-6 [14] and Pim-1 [15] contain AREs and are binding targets for TTP. This suggests that TTP may serve as a tumor suppressor in pancreatic cancer, and TTP dysfunction may promote cancer initiation and progression. However, TTP expression pattern and its role in pancreatic cancer have not yet been reported.

To develop effective therapeutics to treat pancreatic cancer, the molecular mechanisms of tumor initiation and progression must be identified. Therefore, in the present study, a pancreatic cancer tissue microarray including 90 patient tissues was used to examine the relationships between TTP expression and clinicopathologic outcomes. In addition, we investigated the role of TTP in pancreatic cancer cells growth *in vitro* and *in vivo*, and explored the impact of TTP over-expression on tumor related factors. Our results show that TTP over-expression increases apoptosis and reduces cell proliferation in pancreatic cancer, and low TTP expression correlates with poor patient prognosis. Thus, TTP may represent a novel prognostic indicator in pancreatic cancer.

## RESULTS

### TTP expression in clinical pancreatic cancer samples

**Figure 1:**
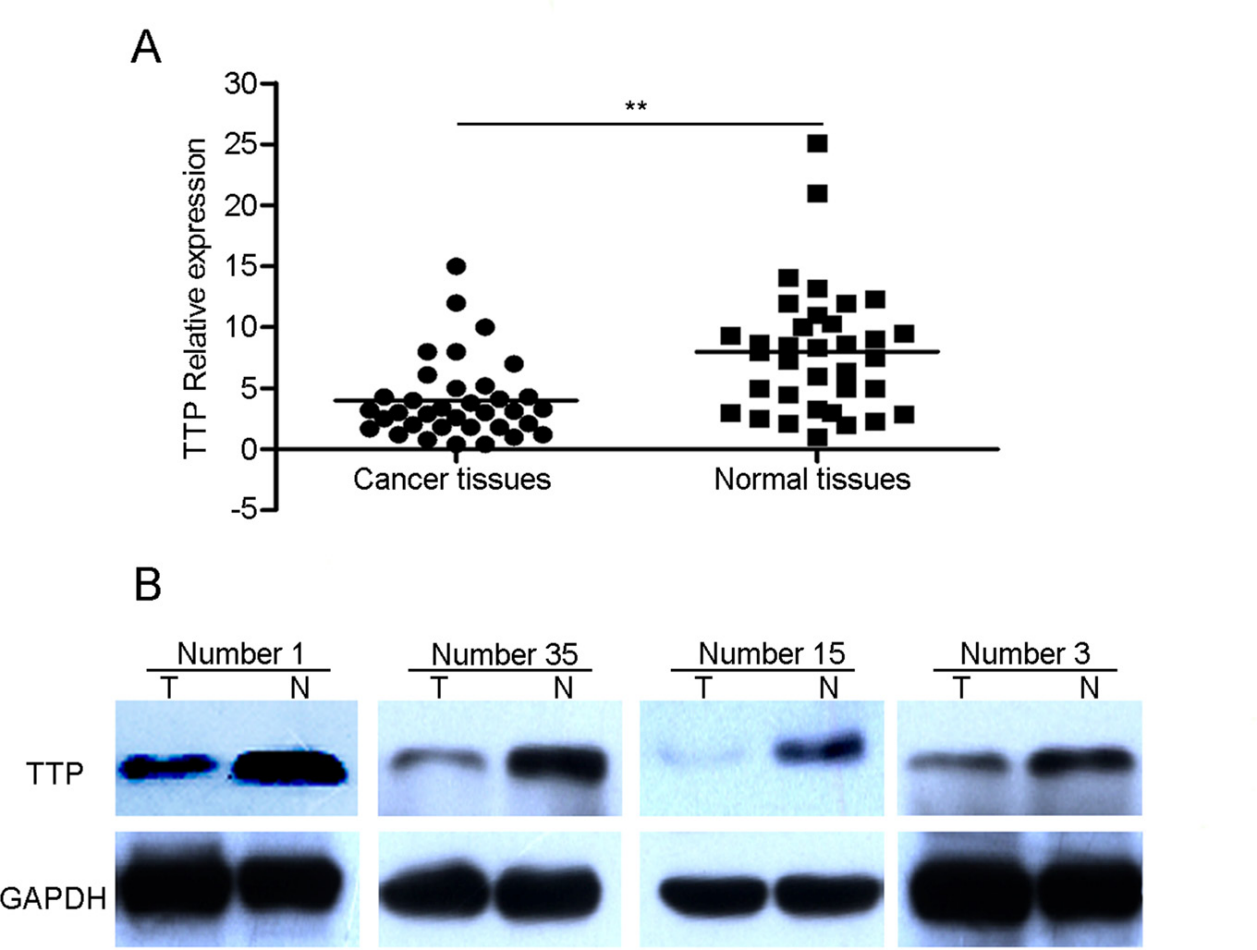
The expression pattern of TTP in patients with pancreatic cancer The average relative expression of TTP mRNA in pancreatic cancer tumor tissues compared to matched adjacent normal tissues (n = 35) **A.** Differential expression of TTP protein in pancreatic cancer tumor specimens compared to matched adjacent normal tissues (n=4) **B.** *P < 0.05, **P < 0.01 vs normal.

To evaluate the expression pattern of TTP in pancreatic cancer and adjacent normal tissues, qRT-PCR was performed to detect mRNA level in 35 paired samples. Compared with paired adjacent normal tissues, TTP mRNA expression was downregulated in 77.1% (27/35) of pancreatic cancer patients (P<0.05)(Figure 1A). TTP protein levels were also lower in pancreatic cancer samples than in matched normal tissue (Figure 1B).

### Correlation between TTP expression and clinicopathologic characteristics in patients with pancreatic cancer

**Figure 2:**
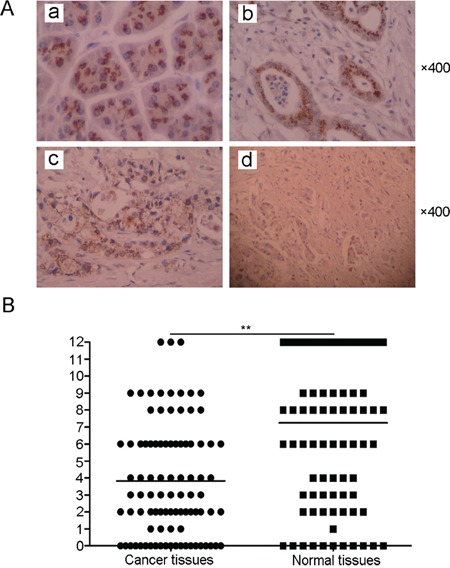
Correlation between TTP expression and clinicopathologic characteristics in patients with pancreatic cancer Normal (non-neoplastic) pancreas **Aa.** Positive TTP expression in well-differentiated tumors **Ab.** Positive TTP expression in moderately-differentiated tumors **Ac.** Negative TTP expression in poorly-differentiated tumors **Ad.** The mean TTP staining score of TTP in pancreatic cancer tumor tissues compared to matched adjacent normal tissues (n = 35) **B.** *P < 0.05, **P < 0.01 VS normal.

To investigate the correlation between TTP expression and pancreatic cancer clinicopathlogic characteristics, tissue microarray blocks were examined using immunohistochemistry. As shown in Figure 2, TTP protein was mainly localized in the cytoplasm. The TTP staining score of tissue microarray blocks was shown in Figure 2B. 71(78.9%) cancer tissue samples showed lower TTP expression than the matched adjacent normal tissues (P <0.01). Low TTP expression was associated with tumor differentiation (P=0.004) (Table 1). Compared to the normal tissues (Figure 2A a), TTP expression was almost negative in patients with poorly differentiated cancer (G3) (Figure 2A d), while weak TTP expression (G2) and strong TTP expression (G1) were found in moderately differentiated pancreatic cancer and well-differentiated pancreatic cancer, respectively (Figure 2A c & 2Ab). In addition, low TTP expression was associated with age(P=0.037), tumor size(P=0.008), pT stage (P<0.001), pN stage (P=0.008) and TNM stage(P<0.001) (Table 1). No significant association was seen between gender, tumor location and TTP expression.

**Table 1:** Pancreatic cancer clinicopathological parameters and their correlation with TTP expression

Features	NO. of patients(%)	Low expression of TTP(%)	P value
**Gender**			
Male	57(63.3%)	47(82.4%)	0.295
Female	33(36.7%)	24(72.0%)	
**Location**			
Head	37(41.1%)	31(83.8%)	0.435
Body and Tail	53(58.9%)	40(75.5%)	
**Age, years**			
<60	41(45.6%)	28(68.3%)	0.037*
>60	49(54.4%)	43(87.5%)	
**Tumor Size, cm**			
<3	40(44.4%)	26(65.0%)	0.008*
>3	50(55.6%)	45(90.0%)	
**Differentiation**			
Well	19(21.1%)	10(52.6%)	0.004*
Moderate/Poor	71(78.9%)	61(85.9%)	
**pT stage**			
T1	16(17.8%)	6(37.5%)	<0.001*
T2	55(61.1%)	48(87.2%)	
T3	19(21.1%)	17(89.4%)	
**pN stage**			
Negative	51(56.7%)	35(68.6%)	0.008*
Positive	39(43.3%)	36(92.3%)	
**TNM stage**			
I	40(44.4%)	24(60.0%)	<0.001*
II-IV	50(55.6%)	47(94.0%)	

* p<0.05, statistically significant.

### Relationship between TTP expression and patient outcome

**Figure 3:**
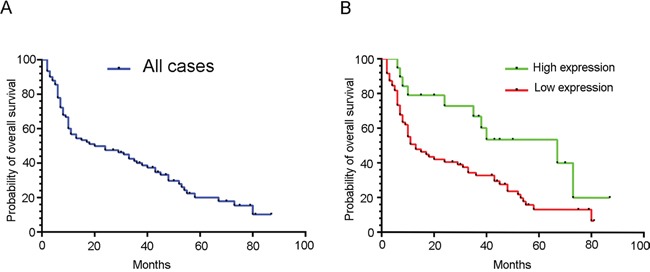
Low TTP expression predicts poor patient outcome Kaplan-Meier curves for overall survival (OS) of 90 pancreatic cancer patients **A.** (B) Kaplan-Meier curves for OS in 90 pancreatic cancer patients with high or low TTP expression **B.**

As we observed, there is a negative correlation between low TTP expression and age, tumor size, pT stage, pN stage, TNM stage, and tumor differentiation. These results suggest that TTP expression is related to the survival rate of patients with pancreatic cancer. To investigate the prognostic value of TTP expression in pancreatic cancer patients, overall survival (OS) analysis was performed in 90 pancreatic cancer cases. We used the Kaplan-Meier method to compare the survival rates of patients with high or low TTP expression. As shown in Figure 3A, the median survival time of all 90 patients with pancreatic cancer was 20 months. Patients with high TTP expression had a median survival time of 67 months, which was significantly longer than the 13 months median survival time observed in patients with low TTP expression (P<0.05) (Figure 3B). From the log rank analysis, patients with high TTP had a longer survival period than those with low expression (P<0.05). Moreover, univariate analysis showed that TTP expression, tumor differentiation, TNM Stage, pT stage, pN stage and tumor size were the factors influencing survival rate in postoperative pancreatic cancer patients (Table 2). Probably because of insufficient sample size, multivariate analysis showed that only AJCC stage was the only independent factor affecting survival rate.

**Table 2:** Cox proportional hazards model analysis of prognostic factors

Variables	Univariate analyses			Multivariate analyses		
	HR	(95%CI)	P value	HR	(95%CI)	P value
**Gender**	**0.774**	**0.468-1.278**	**0.316**			
**Age**	**1.276**	**0.787-2.068**	**0.323**			
**Site**	**0.762**	**0.472-1.231**	**0.267**			
**Tumor Size**	**1.706**	**1.035-2.811**	0.036*	**1.358**	**0.784-2.351**	**0.274**
**Differentiation**	**2.495**	**1.266-4.915**	0.008*	**1.908**	**0.919-3.962**	**0.083**
**pT stage**	**1.845**	**1.258-2.706**	0.002*	**1.504**	**0.964-2.346**	**0.072**
**pN stage**	**2.167**	**1.329-3.534**	0.002*	**0.736**	**0.343-1.579**	**0.431**
**TNM Stage**	**2.944**	**1.762-4.920**	<0.001*	**2.603**	**1.112-6.094**	0.028*
**Low TTP expression**	**2.214**	**1.129-4.340**	0.021*	**0.915**	**0.407-2.058**	**0.831**

* p<0.05, statistically significant.

### TTP over-expression impairs pancreatic cancer cell growth *in vivo* and *in vitro*

**Figure 4:**
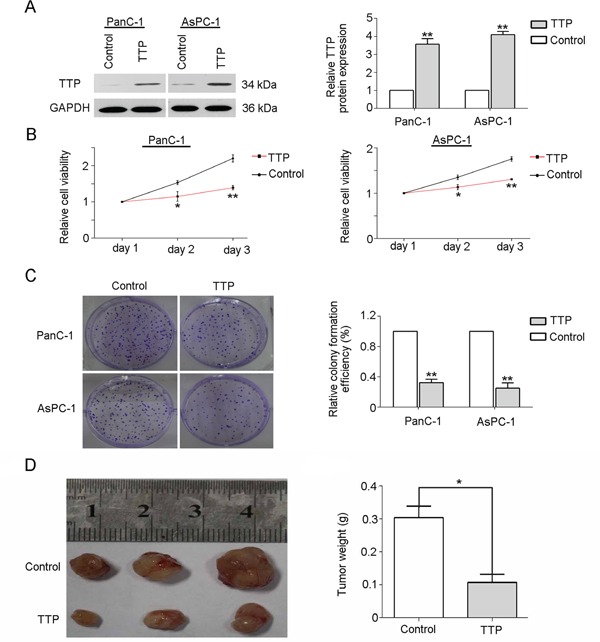
Over-expression of TTP impairs pancreatic cancer cell growth *in vivo* and *in vitro* Pancreatic cancer cell lines (PanC-1 and AsPC-1) were transfected with a TTP over-expression vector and transfection efficiency was confirmed by western blot **A.** TTP over-expression vector-transfected PanC-1 and AsPC-1 cell viability was reduced compared to controls **B.** TTP over-expression reduced colony formation compared to controls **C.** Results from three independent experiments are shown. TTP over-expression reduced tumor weights compared to controls **D.** **P < 0.05, *P < 0.01 VS control.

We found that the expression pattern of TTP was associated with clinicopathologic characteristics and patient survival rate in pancreatic cancer. Tumor cell proliferation rates influence patient prognoses. Some studies reported that TTP over-expression inhibits growth of various cancers, such as malignant glioma [16] and mast cell tumors [17]. These results indicate that TTP likely influences pancreatic cancer patient outcomes via inhibition of proliferation. In order to analyze the effects of TTP on pancreatic cancer cell growth, TTP was over-expressed in PanC-1 and AsPC-1 cell lines. To verify TTP over-expression, whole-cell lysates from pancreatic cancer cell lines transfected with TTP expression vector or control vector were immunoblotted for TTP. As shown in Figure 4A, transfection of PanC-1 and AsPC-1 cell lines with the TTP expression vector resulted in increased TTP levels as compared with cells transfected with control vector. TTP over-expression inhibited PanC-1 and AsPC-1 cell growth, as shown by CCK-8 assays (Figure 4B), and colony formation (Figure 4C) as compared with the controls. TTP over-expression also inhibited tumorigenicity in nude mouse PanC-1 xenografts (Figure 4D, P<0.05).

### TTP over-expression inhibits proliferation and increases apoptosis in pancreatic cancer cells

**Figure 5:**
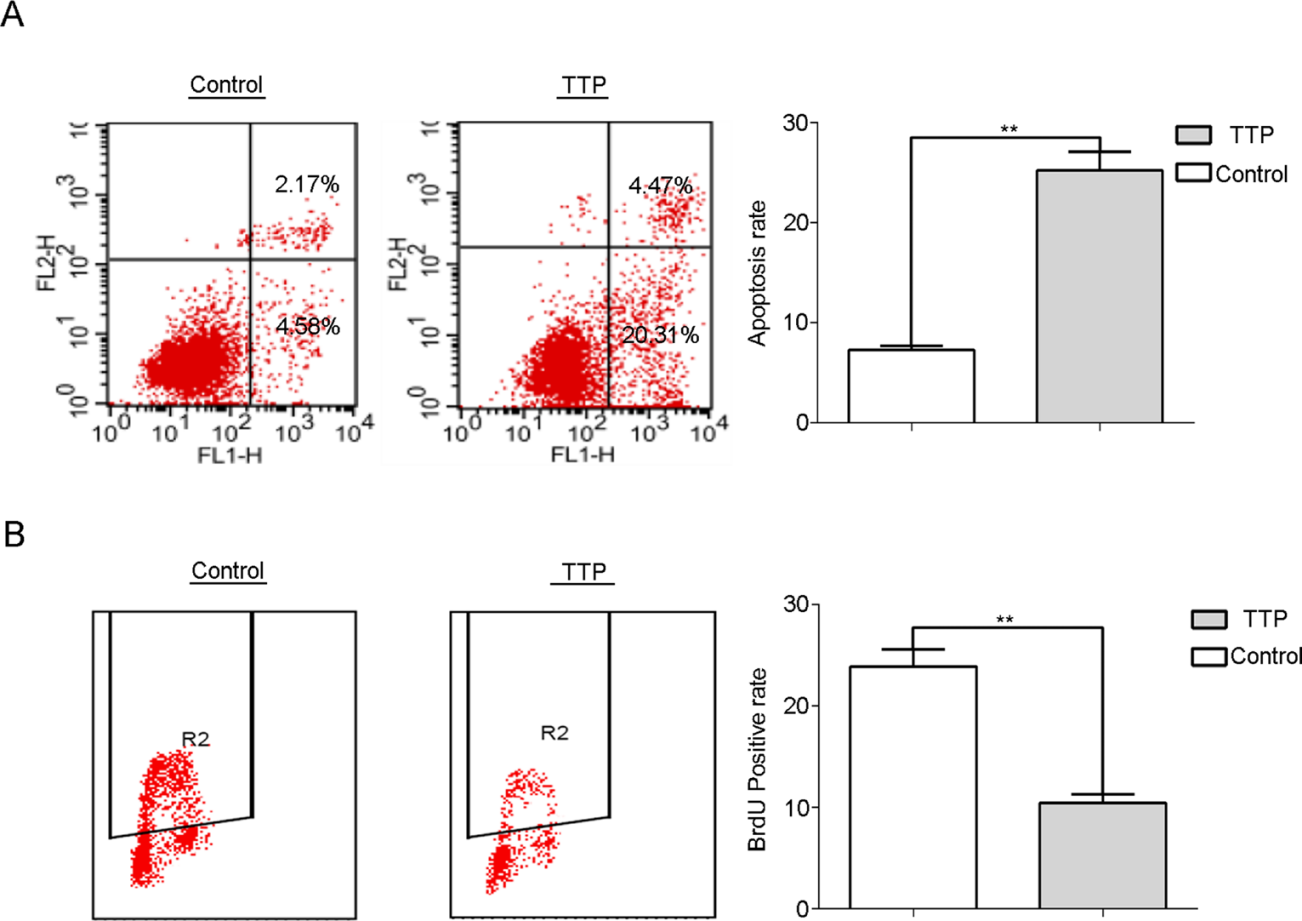
TTP over-expression inhibits proliferation and increases apoptosis in pancreatic cancer cells Apoptosis is increased **A.** and BrdU positivity is decreased **B.** in PanC-1 cells over-expressing TTP compared to the control.

TTP was reported to inhibit proliferation and induce apoptosis in several tumor types, including glioma [18], melanoma [19] and glioblastoma [20]. To explore the mechanism of cell growth inhibition by TTP in pancreatic cancer, FACS analysis was performed on TTP over-expressing and control PanC-1 cells. TTP over-expressing cells displayed higher apoptosis (Figure 5A) and lower BrdU positivity rates (Figure 5B). TTP can thus increase apoptosis and decrease proliferation in pancreatic cancer cell lines.

### RNA-sequencing analysis of TTP over-expressing pancreatic cancer cells

**Figure 6:**
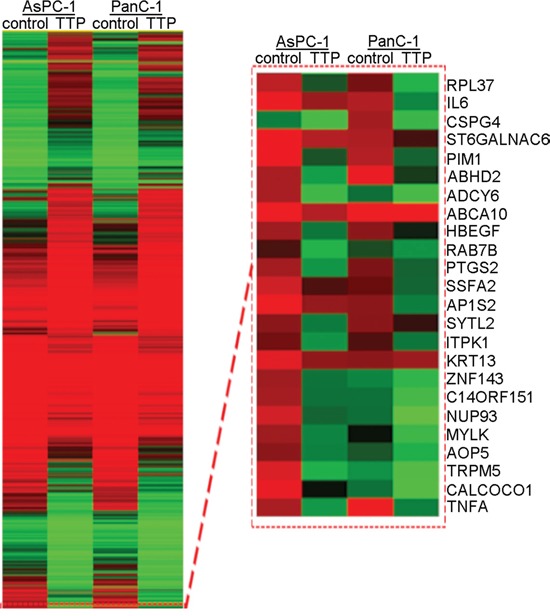
RNA-seq analysis of candidate genes in TTP over-expressing PanC-1 and AsPC-1 cells Expression patterns of RNAs in TTP control/TTP over-expressing PanC-1 and AsPC-1 cells were shown in the left with a heat map (unsupervised clustering). And a selected group of RNAs were also showed in the right with a zoomed view. The data are colored red or green for high abundance/upregulation or abundance/downregulation.

RNA-seq analysis of TTP over-expressing and control PanC-1 and AsPC-1 cells was performed to identify candidate genes with which TTP may interact in pancreatic cancer. Candidate tumor-related factors influenced by TTP over-expression, notably Pim-1 and IL-6, are shown in Figure 6 and Table 3. Given the already-reported roles of Pim-1 and IL-6 in pancreatic cancer tumorigenesis, we speculate that TTP may reduce cancer cell proliferation though downregulation of Pim-1 and IL-6.

**Table 3:** RNA-sequencing intensity data

Gene Name	AsPC-1 Control	AsPC-1 TTP	PNAC-1 Control	PNAC-1 TTP
**CDCA7**	100.34	1525.20	108.10	1341.16
**MMP7**	392.32	4477.66	866.85	17632.18
**B2M**	10206.37	10350.98	11182.79	11631.42
**DNAJC8**	3940.01	4070.18	3412.02	4394.99
**H3F3A**	16600.36	18023.38	15767.12	17644.38
**RPS27**	33459.66	30140.76	32776.52	30049.76
**CCND1**	629.43	2901.49	326.74	4286.73
**ANG**	133.78	487.84	148.90	498.87
**CD47**	155.77	536.93	139.00	613.58
**SERPINB1**	892.50	2946.91	809.61	2171.04
**BOP1**	633.44	2055.89	667.35	2599.78
**ATIC**	648.27	2093.05	366.13	2031.38
**CTXN1**	597.56	1847.56	470.36	1554.73
**CDC2**	302.74	924.50	335.91	1363.50
**MYC**	1694.26	4609.36	1835.38	5707.29
**CEBPA**	168.47	433.56	227.37	534.80
**PCNA**	514.51	1321.44	687.21	1929.87
**PCAF**	214.31	85.18	156.30	72.82
**TNFRSF12A**	3632.25	1404.79	3219.18	1380.99
**CDK6**	3774.33	1386.34	4411.69	1326.97
**PERP**	12366.31	4457.80	12920.83	3674.03
**IL11**	181.99	63.50	186.28	68.61
**CD58**	3705.61	1278.57	4487.06	1294.59
**NFIB**	2423.54	835.35	1194.54	642.34
**MTSS1**	7078.37	2419.14	7044.21	2531.72
**GSPT1**	18299.12	6084.93	21984.91	7539.33
**MSN**	6067.92	2015.14	3155.93	1074.62
**GSK3B**	978.35	322.63	1560.87	395.34
**CD9**	2355.71	772.87	3383.81	803.03
**CYP3A5**	26770.42	7974.92	21481.53	5232.45
**CD55**	2324.96	671.87	3408.34	992.26
**CD59**	1169.64	319.81	979.97	304.61
**CEACAM1**	6670.56	1572.16	4347.23	1333.88
**TGFB3**	317.02	73.69	426.99	59.90
**ESAM**	1495.96	336.17	1613.20	296.97
**MAP3K8**	1785.49	397.40	1247.78	291.10
**IL6**	6059.76	1343.03	22675.16	1074.11
**SGK**	9836.30	2139.78	9348.72	1518.22
**GDF15**	15226.22	2258.03	27176.28	1800.53
**VEGFA**	1922.70	248.10	3915.29	259.74
**CEACAM8**	310.18	38.70	182.50	37.78
**PIM1**	3881.58	251.25	2909.60	272.33

### Pim-1 and IL-6 levels are downregulated in TTP over-expressing cell lines and matched normal tissues

**Figure 7:**
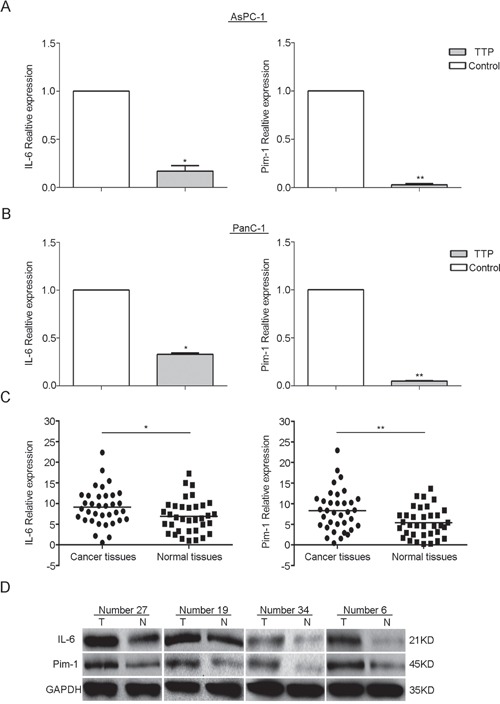
TTP over-expression downregulates Pim-1 and IL-6 in pancreatic cancer cell lines Pim-1 and IL-6 mRNA expression is downregulated in TTP over-expressing PanC-1 and AsPC-1 cells as compared to control cells **A.** and **B.** Pim-1 and IL-6 mRNA was increased in 35 pancreatic cancer tissues as compared to paired normal tissues as shown by qRT-PCR **C.** Pim-1 and IL-6 protein levels were increased in tumor tissues as compared to normal tissues in four randomly chosen matched tissue pairs, as measured by western blot (T = tumor tissue; N = normal tissue) **D.**

qRT-PCR was performed on 35 paired tissues as well as TTP over-expressing PanC-1 and AsPC-1 cells to verify the RNA-seq results. As expected, Pim-1 and IL-6 mRNA levels were significantly reduced in TTP over-expressing PanC-1 (Figure 7A) and AsPC-1 cells (Figure 7B) compared to controls. Conversely, Pim-1 and IL-6 mRNA (Figure 7C) and protein levels (Figure 7D) were increased in pancreatic cancer tissues as compared to normal tissues in four randomly selected paired samples.

## DISCUSSION

Chronic inflammation and high levels of pro-inflammatory gene products are associated with the process of tumorigenesis and can promote cancer cells proliferation, angiogenesis, invasion, survival, chemoresistance, radioresistance and metastasis [21]. Tumor cells produce various chemokines and cytokines as part of the tumor microenvironment, and chronic inflammation is one of the important hallmarks of cancer [22]. Gukovsky, *et al.* suggested that chronic pancreatitis (CP) with persistent low-grade inflammation is a necessary factor in the initiation and progression of pancreatic carcinoma [23]. In pancreatic cancer cell lines, IL-1β promotes cell growth and resistance to chemotherapy [24]. Cytokines attract inflammation-related cell types, including neutrophils, macrophages, mast cells, lymphocytes and others, that produce more cytokines [25]. In the process of inflammation, IL-6 and IL-8 can further strengthen the inflammatory response and induce the production of additional inflammatory cytokines [26]. All these events together support an inflammation-tumorigenesis-inflammation cycle in cancer. Thus, inhibiting inflammation may aid in the prevention of tumorigenesis.

mRNA degradation plays a key role in the regulation of mammalian gene expression, and dysregulation of this process may contribute to expression of various genes associated with excessive inflammation and/or accelerated tumor formation [27]. AU-rich elements (AREs) in the 3′ untranslated region (3′UTR) are important in the programmed degradation of many mRNAs that encode proto-oncogenes and inflammation-promoting proteins [9, 10]. These AREs combine with ARE-binding proteins (ARE-BPs) to promote mRNA decay.

TTP is an ARE-binding protein with the ability to recognize ARE sequences through adjacent “AUUUA” binding sites, and to promote mRNAs degradation through deadenylation [28]. Al-Souhibani, et al. found that TTP downregulates expression of uPA (Urokinase plasminogen activator), uPAR (urokinase plasminogen activator receptor), matrix metalloproteinases 1 and 13 (MMP1 and MMP13) [29] and CXCR4 [30]. CXCR4 was shown to act as a chemoattractant that promotes invasion and migration in breast cancer cells [30]. Our previous studies also demonstrated that TTP decreases expression of MMPs, uPA and uPAR. We showed that TTP regulates many inflammatory and tumor related cytokines, including IL-6, IL-8, TNF-α, COX-2, CCL2 and CCL8, as well as the angiogenesis-related factors VEGF, HIF1 and MKP3 [31].

TTP has been shown by others to play a role in many tumor types. Rounbehler, *et al*. reported that TTP acts as a tumor suppressor protein and demonstrated that TTP suppression is a hallmark of Myc-induced cancers; restoring TTP expression impaired Myc-induced lymphomagenesis [32]. TTP, through downregulation of uPA and uPAR, inhibits U87MG human glioma cell growth [16]. In breast tumor cells, TTP induces cell cycle arrest by targeting the AP-1/c-Jun and NF-κB pathways [33]. TTP mRNA and protein levels were found recently to be significantly decreased in tumors of the colon [34], lung [35], cervix [36], prostate and breast [13]. In our study, we report that TTP expression was significantly reduced in pancreatic tumor samples compared to adjacent normal tissues. TTP expression was almost negative in patients with poorly differentiated cancer, and was weakly positive and highly positive in moderately differentiated and well-differentiated pancreatic cancers, respectively. Low TTP expression was associated with age (P=0.037), tumor size (P=0.008), tumor differentiation (P=0.004), pT stage (P<0.001), pN stage (P=0.008) and TNM stage (P<0.001). Univariate analysis showed that TTP has an independent predictive value for survival in pancreatic cancer patients (P=0.021). TTP over-expression influenced the expression of several tumor-related factors, and our results suggest that TTP may reduce pancreatic cancer cell proliferation and increase patient survival through downregulation of Pim-1 and IL-6. Small sample size was a limitation in our study, and larger prospective studies are needed to confirm our findings. Additionally, the mechanisms that govern TTP expression in pancreatic cancer still need to be addressed. Brook, *et al*. reported that the p38 Mitogen-Activated Protein Kinase (p38 MAPK) pathway regulates the stability and localization of TTP [37]. Though RNA-sequencing analysis we identied several candidate genes, mostly inflammation-related, that may be regulated by TTP expression in pancreatic cancer. However, the effects of TTP on the downstream signaling pathways in pancreatic cancer are still unknown, and more in-depth molecular mechanism research will be carried out in the future. In summary, we found that TTP inhibits cell growth and increases apoptosis in pancreatic cancer. Low TTP expression was correlated with low patient survival rates and poor prognoseis. These results suggest that TTP could act as a prognostic indicator in pancreatic cancer.

## MATERIALS AND METHODS

### Ethics statement

This study was approved through the Ethics Committee of the Scientific and Ethical Committee of Second Military Medical University (SMMU). In addition, informed consent form was received from all participants.

### Patient specimens

All tissue specimens including 90 pancreatic cancer tissues and their matched normal pancreatic tissues, were obtained at surgery from the Shanghai Changzheng hospital. All noncancerous human pancreatic tissue samples were obtained from resection of adjacent pancreatic cancer margins greater than 5 cm. All patients underwent resection and were not treated with other relative treatment such as chemotherapy or radiotherapy.

### Immunostaining and evaluation

Immunohistochemical staining was performed using the EnVision system (Dako Carpinteria, USA). TTP expression in the sections was detected with a primary antibody against TTP (ab119779, Abcam, USA). Immunohistochemical staining was independently analyzed by two pathologists who were blinded to each other's findings. Evaluation of immunostaining was conducted as described previously [38]. Briefly, the staining intensity of TTP was scored semi-quantitatively by analyzing the mean signal intensity (0=negative expression, 1=weak expression, 2=moderate expression, 3=strong expression). Meanwhile, the percentage of TTP positive expression tumor cells was assessed and a proportion score was ascribed to TTP (0 if 0-5%, 1 if 5-25%, 2 if 26-50%, 3 if 51-75% and 4 if 76-100%). The staining intensity scores and proportion scores were then multiplied to give an overall score. For each sample pair, TTP expression considered low when the composite score in cancer tissues less than or equal to that in normal tissues. On the contrary, when cancerous tissue displayed a higher composite score than adjacent normal tissue, TTP expression was considered high.

### Cell culture

PanC-1 and AsPC-1 cell lines were provided by the Institute of Biochemistry and Cell Biology of the Chinese Academy of Science. All cells for experiment were cultured in Roswell Park Memorial Institute 1640 medium (RPMI-1640, Biowest, French) supplemented with 10% FBS (Biowest, French), 100 U/mL penicillin, 100 μg/mL streptomycin sulfate and 1 mmol/L sodium pyruvate at 37°C in 5% CO_2_. The cells were passaged using trypsin-EDTA (Gibco, USA).

### Cell transfection

The TTP over-expression vector (pCMV6-AC-GFP) was purchased from OriGene (RG202049, USA). Cells were subcultured at 1×10^5^ cells per well in 6-well tissue culture plates. After 24 h, cells were transfected with vector using Lipofectamine 2000 (LP 2000) Transfection Reagent (Invitrogen, USA). LP 2000 and vectors werediluted in Opti-MEM medium (Gibco, USA) independently and incubated for 15 min. Subsequently, 100ul Opti-MEM containg 8ul LP2000 and 100ul Opti-MEM containg 2ug vector was mixed and incubated for 25 min. Finally, the LP2000-vector-Opti-MEM mixture was added in Opti-MEM medium, the final overall volume was 2ml. The medium was changed after 6 h, and cells were incubated for 24-48 h. Cells were then selected with G418 (Gibco, USA) for 48 h. G418-resistant clones were generated and screened using western blot analysis.

### qRT-PCR analysis

Total RNA was isolated from fresh tissues and cultured cells using a standard TRIzol protocol (Invitrogen, USA. RNA quality (A260/A280 ratio) and quantity were assessed by spectrophotometry. 1mg of total RNA was used for cDNA synthesis using the RevertAidtm First Strand cDNA Synthesis Kit #1622 (Fermentas, Lithuania) according to the manufacturer's protocol. Appropriate forward and reverse primers were used for the reverse transcriptase polymerase chain reactions (RT-PCR) to detect the transcripts of interest. The primer sequences for GAPDH, TTP, IL-6 and Pim-1 have been previously described [39, 40, 41]. The PCR conditions were as follows: 94°C for 10min, then 40 cycles of 94°C for 30 sec, 55-58°C for 30 sec, and 72°C for 45 sec, followed by 72°C for 10 min. qRT-PCR was performed using a 7300 Real-time PCR System (Applied Biosystems, USA). Standard curves were plotted for each optimized assay, each of which produced a linear plot of the threshold cycle (Ct) against log (dilution). Each gene was quantified based on the concentration obtained from the standard curve and was presented in arbitrary unit (AU). The quantity of each gene was normalized against GAPDH.

### Western-blot analysis

Cultured cells and crushed human tissue specimens were lysed in buffer containing 50 mol/L Tris(hydroxymethyl)aminomethane-HCL (Tris-HCl, pH = 6.8), 2% sodium dodecyl sulfate (SDS), 10% glycerol, phosphatase inhibitors (100 mmol/L Na3VO4, 10 mmol/L NaF) and 1 mmol/L phenyl methylsulphonyl fluoride (PMSF, Beyotime, Shanghai) to obtain whole cell lysates. Insoluble debris was removed by centrifugation at 13,000g and 4°C for 15 min. The supernatant was collected. Total protein concentration was determined using a BCA protein assay reagent (Beyotime, Shanghai). Proteins were separated by SDS-PAGE, then transferred to a nitrocellulose membrane (NC membrane), incubated with 10% skimmed milk at room temperature for 1h, then incubated with antibodies against TTP (ab119779, Abcam, USA), IL-6 (sc-7920, Santa Cruz, USA), Pim-1 (ab117110, Abcam, USA) or GAPDH (2118, cell signaling, USA) at 4°C overnight. The immunocomplexes were visualized using a horseradish peroxidase-conjugated (HRP-conjugated) antibody followed by incubation with the chemiluminescence reagent (Millipore, USA) and exposure to a photographic film. Western blot results were processed and analyzed using Quantity One software (Bio-Rad, USA).

### Cell proliferation assay

The cells (1×10^4^/mL) were plated onto 96-well plates. At 24, 48 and 72 h post-transfection with TTP over-expression or control vector, cell viability was analyzed by CCK-8 assay. The experiments were performed according to the manufacturer's protocol in triplicate.

### Colony formation assay

Colony formation assays were performed as previously described [42]. Briefly, the cells (500/well) were cultured in 6-well plates for 2 weeks. The colonies were counted and photographed using Quantity One software (Bio-Rad, USA). The experiments were performed in three times.

### Tumorigenicity assay in nude mice

The tumor formation abilities of TTP over-expressing pancreatic cancer cells were evaluated by injecting cell suspensions into BALB/c nude male mice. Ten mice were randomly divided into TTP over-expressing and control group. For each mouse, 2×10^7^ cells of PanC-1 cells (stably transfected with the TTP expression vector or control vector) were injected into the buttocks. After 4-5 weeks, mice were sacrificed and the tumor weighed.

### Cell apoptosis and proliferation

Harvested cells were washed twice with PBS. After centrifugation, buffer was added to bring the concentration to 1×10^6^ cells/ml. Cells were then incubated with Annexin V and Propidium iodide (PI) for 10 min at room temperature, protected from light. Apoptosis and 5′-bromo-deoxyuridine (BrdU) incorporation were detected by FACS. Cells were exposed to BrdU for 1 h, after which cell proliferation was quantified using the BrdU Cell Proliferation Detection kit (Keygentec, China).

### RNA isolation and purification

Total RNA from TTP over-expressing and TTP control PanC-1 cells was extracted and purified by the RNeasy Mini Kit plus D Nase-I treatment (QIAGEN, China) in accordance with the manufacturer's instructions. An Agilent 2100 Bioanalyzer (Agilent Technologies, USA) was used to evaluate RNA quality.

### RNA sequencing

RNA sequencing was performed on TTP over-expressing and TTP control PanC-1 and AsPC-1 cells as previously reported [43]. Briefly, RNA integrity number (RIN) ≥ 7.0, protein- and phenol-free RNA samples were used for the microarray analysis. Using 101-bp pair-end sequencing strategy, sequencing libraries were sequenced on Illumina HiSeq 2000 platform (Illumina, USA).

### Statistical analysis

Statistical analyses were performed using SPSS 13.0 software. Data were expressed as means ± SD. Differences between mRNA detection, H-score and western blotting results were assessed by paired t-test. Pearson's X^2^ test was applied to analyze the association of TTP expression with clinipathological parameters. Kaplan-Meier analysis and log-rank tests were performed to assess survival rate and to compare differences in survival curves. Cox regression analysis was used to assess the significance of multiple survival predictors. Relative cell viability, relative colony formation efficiency, tumor weight, apoptosis rate, BrdU positive rate and RNA-seq were analyzed using Student's t-test. Differences were considered significant at p < 0.05.
